# Optical Properties of GePb Alloy Realized by Ion Beam Technology

**DOI:** 10.3390/ma18102258

**Published:** 2025-05-13

**Authors:** Shuyu Wen, Yuan-Hao Zhu, Oliver Steuer, Mohd Saif Shaikh, Slawomir Prucnal, René Hübner, Andreas Worbs, Li He, Manfred Helm, Shengqiang Zhou, Jun-Wei Luo, Yonder Berencén

**Affiliations:** 1 State Key Laboratory of Semiconductor Physics and Chip Technologies, Institute of Semiconductors, Chinese Academy of Sciences, Beijing 100083, China; sywen@semi.ac.cn (S.W.); zhuyuanhao20@semi.ac.cn (Y.-H.Z.); heli2018@semi.ac.cn (L.H.); 2 Helmholtz-Zentrum Dresden-Rossendorf, Institute of Ion Beam Physics and Materials Research, Bautzner Landstrasse 400, 01328 Dresden, Germany; o.steuer@hzdr.de (O.S.); m.shaikh@hzdr.de (M.S.S.); s.prucnal@hzdr.de (S.P.); r.huebner@hzdr.de (R.H.); a.worbs@hzdr.de (A.W.); m.helm@hzdr.de (M.H.); s.zhou@hzdr.de (S.Z.); 3 Center of Materials Science and Optoelectronics Engineering, University of Chinese Academy of Sciences, Beijing 100049, China; 4 Institut für Werkstoffwissenschaft und Max-Bergmann-Zentrum für Biomaterialien, TUD Technische Universität Dresden, 01062 Dresden, Germany; 5 Faculty of Electrical and Computer Engineering, Technische Universität Dresden, Helmholtzstraße 18, 01069 Dresden, Germany; 6 Institute of Applied Physics, Technische Universität Dresden, 01062 Dresden, Germany

**Keywords:** ion implantation, GePb, bandgap engineering, photoluminescence

## Abstract

Incorporating lead (Pb) into the germanium (Ge) lattice emerges as a promising approach for bandgap engineering, enabling luminescence at longer wavelengths and paving the way for enhanced applications in short-wave infrared (SWIR) light sources and photodetectors. In this work, we report on optical properties of GePb alloys fabricated by a complementary metal-oxide semiconductor (CMOS)-compatible process that includes Pb ion implantation followed by solid-phase epitaxial regrowth via flash-lamp annealing. Optical characterization, including photoluminescence spectroscopy and Fourier-transform infrared reflectance spectroscopy, reveals that GePb alloys exhibit a reduced bandgap compared to pure Ge, resulting in longer-wavelength emission, while also providing broadband antireflective properties below 1800 nm wavelengths due to the surface subwavelength nanostructure. These findings position nanostructured GePb as a highly promising candidate for SWIR optoelectronic applications.

## 1. Introduction

The bandgap engineering of germanium (Ge) can be achieved by ultra-high n-type doping [1,2,3,4,5], introducing tensile strain [6,7] or alloying Ge with larger group-IV atoms, such as tin (Sn) [8,9,10,11,12,13]. These approaches modify the band structure, reducing the bandgap [14], thereby extending Ge emission to longer wavelengths [15] and significantly enhancing its potential for short-wave infrared (SWIR) optoelectronic applications.

Currently, alloying Ge with Sn has achieved great success in SWIR applications, including extended infrared GeSn photodetectors [16,17,18,19] and GeSn light sources [15,20,21], paving the way for on-chip optoelectronic integration. Similar to GeSn alloys, incorporating lead (Pb) into the Ge lattice can also reduce the bandgap and extend the optoelectronic applications to longer wavelengths. Due to its larger atomic radius compared to Sn [22,23], Pb induces a stronger bandgap modification when incorporated into the Ge lattice. First-principle calculations indicate that incorporating just 3.4% Pb can transform Ge into a direct-band structure, and higher Pb concentrations will further shift the absorption edge into the mid-infrared range [24,25,26]. Additionally, simulations show that the optical gain of GePb alloy increases abruptly with higher Pb concentration, indicating great promise in on-chip light-source applications [26].

However, obtaining GePb alloys with high Pb content remains challenging due to the limited equilibrium solid solubility of Pb in the Ge lattice, which is less than 0.5% at room temperature, thus requiring non-equilibrium fabrication methods [27,28]. Furthermore, the large atomic radius difference (23.2%) between Ge and Pb atoms limits the material quality of the regrowth of the GePb layer on the Ge substrate during the annealing process [29]. Ion implantation is a typical method to achieve non-equilibrium doping or alloying above the solid solubility. However, the high defect density in the implanted and annealed material significantly enhances nonradiative recombination, which quenches the photoluminescence (PL) [30,31] and limits optoelectronic applications.

In this work, we investigate the optical properties of GePb alloys, featuring a broadband antireflective subwavelength nanocavity layer using a CMOS-compatible process based on ion implantation combined with ultra-fast flash-lamp annealing (FLA). The Pb concentration of our GePb alloy is estimated to be 0.7%. While lead-free technologies are preferred in many CMOS applications, the minute concentrations of Pb in our GePb alloys minimize contamination concerns. We observed room-temperature PL in the GePb alloys, with spectral extension beyond 1900 nm, indicating low amounts of non-radiative defects [32] and broadband antireflection properties of GePb samples ascribed to the antireflective subwavelength nanocavity layer. We performed detailed characterization of the bandgap engineering through low-temperature PL measurements comparing these results with a reference Ge-virgin sample. PL emission from the GePb alloys was detectable from 1800 nm to 2060 nm at liquid nitrogen temperature (77 K). This work serves as a proof-of-concept for bandgap engineering in GePb materials. With wavelength-extended photoluminescence and broadband antireflection properties, the GePb material holds promise for SWIR optoelectronic applications.

## 2. Materials and Methods

The Ge wafer used in this work was a commercial intrinsic 4-inch (100) Ge wafer with a resistivity of 50 Ω·cm and a thickness of 300 μm. This double-side polished wafer was capped with a 40 nm thick SiO_2_ layer deposited via plasma-enhanced chemical vapor deposition (PECVD) before ion implantation. Pb ions were implanted into the Ge wafer at a fluence of 2 × 10^15^ cm^−2^ and an energy of 200 keV at room temperature, resulting in the formation of a GePb-implanted layer beneath the surface. During the ion implantation, the spot size of the ion beam was 5 mm × 5 mm. After Pb ion implantation, the GePb wafer was cut into 10 mm × 10 mm for subsequent flash-lamp annealing (FLA). We performed FLA on the implanted GePb samples in a continuous flow of N_2_ with firstly preheating at 180 °C for 60 s (heating rate of 3 K/s) and subsequently a flash with an energy density of 78 J/cm^2^ for 3.2 ms (heating rate of 10^5^ K/s). The wafer temperature during FLA is estimated to be approximately 50 °C below the melting point of Ge. This estimation is based on the energy density of the flash (78 J/cm^2^) and the heating conditions, which were optimized to achieve solid-phase epitaxial regrowth without reaching the melting point of Ge.

The Raman scattering spectra were recorded in the backscattering geometry of HORIBA micro-Raman system using 100× objective (Olympus, Hachioji-shi, Japan), 532 nm pump laser (HORIBA Ltd., Palaiseau, France), and HORIBA Syncerity Si-CCD detector (HORIBA, Kyoto, Japan). Reflectance spectra were recorded in the Bruker VERTEX 70v Fourier-transform infrared (FTIR) spectroscopy system (Bruker, Billerica, MA, USA) with a Au mirror as the reference of 100% reflection. Photoluminescence (PL) spectra were recorded in a home-made micro-PL system equipped with a 532 nm laser, a 50× visible-infrared objective (Olympus, Hachioji-shi, Japan), and the HORIBA Symphony II InGaAs-CCD detector (HORIBA, Kyoto, Japan). Rutherford backscattering spectrometry (RBS) was carried out using the 1.7 MeV He^+^ beam of the Rossendorf van de Graff accelerator (Ion Beam Center, Helmholtz-Zentrum Dresden-Rossendorf, Dresden, Germany).

To characterize the microstructure of the GePb sample, bright-field transmission electron microscopy (TEM) imaging and high-resolution TEM imaging were performed with an image-*C*_s_-corrected Titan 80–300 microscope (FEI, Eindhoven, The Netherlands) operated at an accelerating voltage of 300 kV. Prior to TEM analysis, the specimen mounted in a high-visibility low-background holder was placed for 8 s into a model 1020 Plasma Cleaner (Fischione, Export, PA, USA) to remove potential contamination. Cross-sectional TEM lamella preparation was performed by in situ lift-out using a Helios 5 CX focused ion beam (FIB) device (Thermo Fisher, Brno, Czech Republic). To protect the sample surface, a carbon cap layer was deposited via electron-beam-induced deposition, followed by Ga-FIB-assisted precursor decomposition. Afterwards, the TEM lamella was prepared using a 30-keV Ga-FIB with adapted currents. Its transfer to a 3-post copper lift-out grid (Omniprobe, High Wycombe, UK) was performed with an EasyLift EX nanomanipulator (Thermo Fisher, Brno, Czech Republic). To minimize sidewall damage, Ga ions with only 5-keV energy were used for the final thinning of the TEM lamella to electron transparency. Top-down scanning electron microscopy (SEM) imaging of the flash-lamp-annealed GePb sample was performed with an S-4800 microscope (Hitachi, Tokyo, Japan) operated at an accelerating voltage of 5 kV.

## 3. Results and Discussion

**Figure 1:**
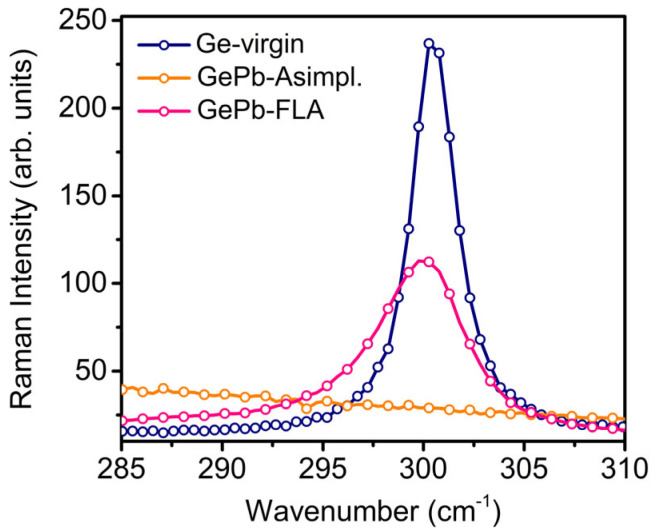
Raman measurement spectra of reference Ge-virgin sample, as-implanted (Asimpl.) GePb, and flash-lamp-annealed GePb samples.

Figure 1 displays the micro-Raman spectra obtained from virgin Ge, as-implanted GePb, and flash-lamp-annealed GePb samples. The main peak near 300.5 cm^−1^ in the reference Ge-virgin sample corresponds to the Ge-Ge transverse optical (TO) phonon mode. It depends on the Ge-Ge bond length of the Ge lattice, which can be affected by the presence of alloying atoms and the strain conditions within the Ge lattice [4]. After Pb ion implantation, the TO phonon mode of Ge disappears, indicating an amorphous structure due to implantation-related crystal damage. In the FLA-treated GePb sample, the TO phonon mode is located at 300.0 cm^−1^, showing a slight shift toward lower wavenumbers compared to pristine Ge. This shift is attributed to the increased Ge-Ge bond length due to the larger Pb atoms incorporated into the lattice. The full width at half maximum (FWHM) of the TO phonon mode in the FLA-treated GePb sample is 5.5 cm^−1^, significantly broader than that of pristine Ge (2.7 cm^−1^), indicating the presence of residual lattice damage in the annealed GePb layer.

**Figure 2:**
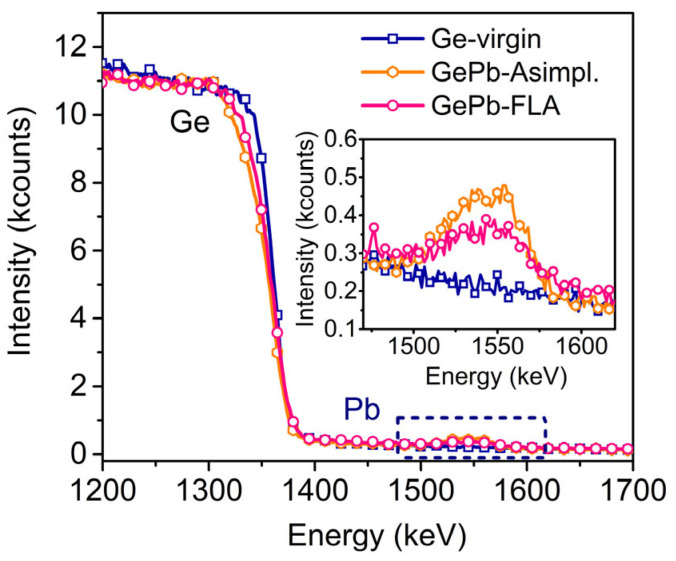
The results of RBS measurement along the random direction of reference Ge-virgin, as-implanted GePb, and flash-lamp-annealed GePb samples, while SiO_2_-capped layers of all of the samples are removed before RBS measurement.

We utilized Rutherford backscattering spectrometry (RBS) in a random direction to confirm successful Pb implantation and analyze the distribution of Pb before and after the annealing process (see Figure 2). The SiO_2_ capping layer was removed by HF etching before RBS measurement. In both the as-implanted (Asimpl.) and FLA-treated GePb samples, Pb contribution appears as a peak in the spectra at an energy between 1450 and 1600 keV. The Pb profile shows an intensity decrease after FLA, which indicates the loss of the Pb element in the annealed sample. This can be ascribed to the flash-lamp annealing process being carried out on a millisecond timescale and the Pb alloying concentration exceeding the solid solubility. During the annealing process, Pb atoms can redistribute, resulting in an overall decrease in concentration. Some of these Pb atoms may diffuse to the surface and are subsequently removed by HF etching. As demonstrated by Raman spectroscopy, the Pb-implanted Ge layer becomes crystalline. The effective crystallization during the FLA process was later confirmed by a cross-sectional TEM study. Unfortunately, the actual Pb concentration cannot be calculated using the RBS profile due to the porous surface formed during ion implantation. However, based on simulations using the Stopping and Range of Ions in Matter (SRIM) software (SRIM-2013) [33], combined with RBS measurements, we estimate the nominal Pb concentration to be approximately 0.7%. Further details on the SRIM simulation and Pb concentration estimation are provided in the Supplementary Materials.

**Figure 3:**
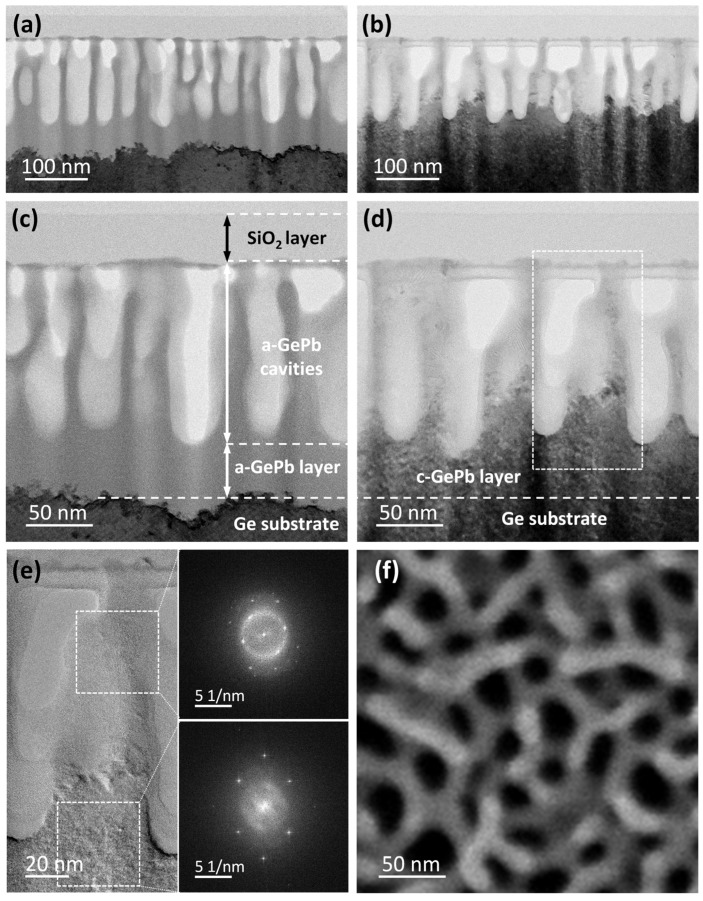
(**a**,**b**) Overview bright-field TEM images and (**c**,**d**) magnified views of as-implanted GePb (**a**,**c**) and flash-lamp-annealed GePb (**b**,**d**). The featureless columns in the image indicate the cavity structure, while the light gray and dark gray areas represent the amorphous GePb (a-GePb) and crystalline GePb (c-GePb), respectively. It should be noted that the contrast variations within and below the cavity structure are not due to the Pb implantation but, rather, are caused by different thinning rates during focused-ion-beam-based preparation of the TEM lamellae. (**e**) High-resolution TEM image (left) for the rectangular region marked in panel (**d**) together with the fast Fourier transforms (right) for the two quadratic regions. (**f**) SEM image showing the surface morphology of the flash-lamp-annealed GePb sample. The SiO_2_ capping layer was removed before SEM measurement.

Figure 3a–e summarize cross-sectional transmission electron microscopy (TEM) results of the as-implanted GePb and flash-lamp-annealed GePb samples. In particular, the overview bright-field TEM image in Figure 3a and the magnified view in Figure 3c show that, after Pb ion implantation, the GePb surface structure is fully amorphous, with a clear interface between the amorphous GePb-implanted layer (appearing light gray) and the single-crystalline Ge substrate (appearing dark gray). Regarding morphology, the GePb layer is not homogeneous but shows a cavity structure within the first 130 nm below the SiO_2_ capping layer and a 60 nm homogeneous amorphous GePb layer between this cavity structure and the single-crystalline Ge substrate. Based on the TEM images, we estimate that the maximum implantation depth of Pb reaches approximately 190 nm below the surface. The relatively high atomic weight of Pb and the high implantation energy result in the formation of the cavity-rich surface structure during implantation.

This typical surface morphology can also be observed in Ge implanted with heavy ions (such as Mn [34], In [35], and Sb [36]) at room temperature and arises from the formation and accumulation of vacancy-related clusters, which minimize the dangling bond density [37,38]. During the ion implantation process, the ion beam collides with the Ge lattice, creating a vacancy-rich region close to the surface. Vacancy clustering in this near-surface region accelerates ion collisions with the Ge substrate [39], leading to the growth of cavity structures perpendicular to the sample surface [40]. However, there is not yet a conclusive explanation for the various nanostructures observed in Ge upon implantation [41]. Furthermore, Ref [42] demonstrates the potential to enhance the ordering of self-organized surface nanostructures and achieve large-area uniformity by increasing the implantation fluence.

Similar surface morphology is also observed in GeSn alloys fabricated through room-temperature ion implantation [43,44,45]. However, pulsed-laser annealing destroys these cavity structures due to the melting and liquid-phase epitaxial regrowth process, hindering the realization of group-IV Ge alloys with surface morphology. In this work, Pb’s significantly higher atomic weight compared to Sn leads to the formation of high-aspect-ratio cavity structures. In addition, the ultra-fast solid-phase epitaxial regrowth achieved by FLA preserves these high-aspect-ratio cavity structures during recrystallization.

As illustrated in Figure 3b,d, the flash-lamp annealing process yields solid-phase epitaxial regrowth for the 60 nm homogeneous amorphous GePb layer. However, for the sidewalls of the GePb cavity structure (shown in detail in Figure 3e), epitaxial regrowth occurs only in the lower quarter up to one-third, where the diffractogram indicates a single-crystalline GePb structure. In contrast, the upper cavity sidewalls exhibit a polycrystalline structure, indicating random nucleation during the regrowth process. Such random nucleation may be due to an inhomogeneous temperature distribution within the cavity structure, which might have a faster cooling rate than the GePb layer, and the absence of regrowth seeds during the recrystallization process [46], thus resulting in only polycrystal formation in this region (Figure 3e). High-resolution TEM images of the as-implanted and FLA-treated GePb samples, highlighting the clear contrast between the amorphous layer and the epitaxially regrown crystalline layer, are presented in the Supplementary Materials (Figure S2). The surface morphology of the FLA-treated sample is shown in the top-view SEM image in Figure 3f. While the black regions in the SEM image correspond to cavities in the GePb surface structure, the bright regions represent the corresponding side walls. While TEM provides a clear view of the nanocavity distribution and crystal structure in the vertical direction, it is limited in revealing the lateral distribution of the nanocavities. This is because the FIB-prepared sample is laminar, making it difficult to observe details such as the shape and diameter distribution of the nanocavities. In contrast, the top-view SEM image offers a comprehensive perspective on these lateral characteristics, providing valuable complementary information.

Here, we achieved partial solid-phase epitaxial recrystallization using an ultra-fast FLA process, which occurs on a millisecond scale. This allowed us to attain recrystallization while preserving the surface nanostructure. This surface morphology, featuring cavity structures with diameters in the tens of nanometers, exhibits antireflective properties over a broadband wavelength range, as confirmed by following optical characterization, positioning GePb as a promising material for optoelectronic applications.

**Figure 4:**
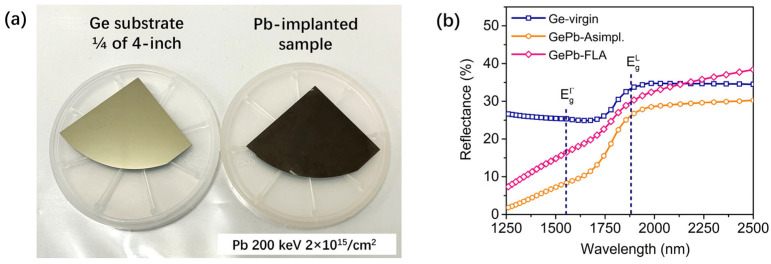
(**a**) Image of Ge-virgin substrate (left) and 200 keV 2 × 10^15^/cm^2^ Pb-implanted sample (right). After implantation, the GePb sample exhibited a dark color visible to the naked eye, indicating broadband antireflective properties. (**b**) Reflectance spectra of Ge-virgin, as-implanted GePb, and flash-lamp-annealed GePb samples. The direct bandgap and indirect bandgap of Ge are shown as Eg^Γ^ and Eg^L^. The decreased reflectance in the GePb samples indicates broadband antireflective properties due to the cavity structures.

After implantation, the GePb sample exhibited a dark color visible to the naked eye (Figure 4a, right), indicating broadband antireflective properties of subwavelength nanostructures led by Pb implantation. In contrast, the polished Ge reference wafer (Figure 4a, left) shows high reflectance in the visible range. Figure 4b shows the optical reflectance spectra of the Ge-virgin sample, as-implanted GePb, and flash-lamp-annealed GePb samples. The Ge-virgin reference sample exhibits a characteristic reflection increase at the edge of its bandgap, attributed to backscattered light from the back surface of the Ge substrate and high transmission below its energy bandgap [47]. Following Pb implantation, a significant decrease in reflectance for wavelengths shorter than the bandgap energy was observed, indicating broadband antireflective properties in the GePb samples. After flash-lamp annealing, the reflectance remained substantially lower than that of the Ge-virgin sample within the same wavelength range, suggesting successful preservation of the cavity surface structure during solid-phase epitaxial regrowth via flash-lamp annealing.

In this work, the size of our nanostructures is much smaller than the wavelength, and the mechanism differs from typical “light trapping” structures [48,49], which generally have features on the order of the wavelength. The antireflective effect in the GePb sample can be attributed to the smooth gradient in the refractive index at the air–substrate interface, facilitated by the subwavelength nanocavity structure on the GePb surface (as shown in Figure 3). Polished Ge wafers exhibit a reflectance exceeding 35% across wavelengths of 350–1800 nm, primarily due to the significant refractive index contrast between Ge (refractive index ~4) and air (refractive index ~1), which causes Fresnel reflections at the polished surface [47]. Subwavelength nanostructures mitigate this issue by creating a smooth refractive index gradient at the interface [50,51], allowing the surface to function as a broadband antireflective layer on germanium [52,53]. In this work, the subwavelength nanocavity structure induced by Pb implantation enables the GePb surface layer to function effectively as a broadband antireflective layer. Such a layer reduces reflectance from the Ge surface and enhances device performance in optoelectronic applications, including photodetectors [54] and solar cells [47]. While the primary goal of adding Pb is to shrink the bandgap and extend the optical activity into the SWIR region, the synergistic effect of reduced reflectance and bandgap modification makes GePb a promising candidate for optoelectronic applications.

However, characterizing the bandgap engineering of GePb using absorptance data, which combines reflectance and transmission measurements, is highly challenging for our GePb material. This difficulty arises primarily due to the overlapping absorption between the GePb layer and the Ge substrate. The GePb layer is only 200 nm thick, whereas the Ge substrate is 300 μm thick and exhibits strong absorption below the bandgap. The Ge substrate has a transmission of less than 40% beyond 2 μm, likely caused by sub-bandgap impurity absorption, as reported in Ref. [55]. This overlap complicates isolating the absorption signature of the implanted layer.

Typically, high-fluence ion implantation results in a high concentration of defects in the material, which are challenging to remove during subsequent ultra-fast annealing processes. These remaining detects strongly contribute to nonradiative recombination, limiting the application of high-fluence implantation in light source applications. In this work, we successfully conducted PL measurements on the FLA-treated GePb sample under 532 nm laser excitation, and a new emission peak can be observed under 77 K, which might indicate the bandgap engineering of Ge realized by Pb alloying.

**Figure 5:**
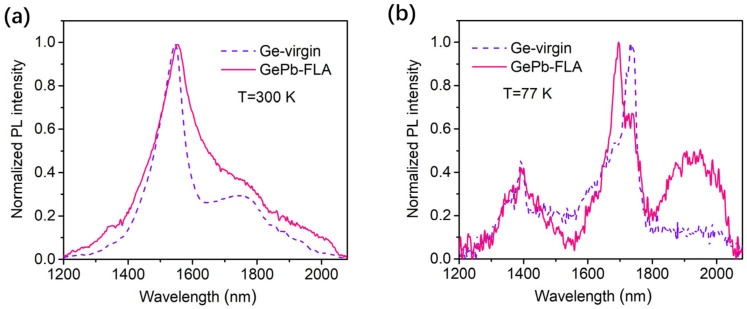
Normalized PL spectrum comparison of Ge-virgin and GePb-FLA samples at room temperature (**a**) and 77 K (**b**). The spectra show that the PL signal of the GePb-FLA sample has contributions from both the GePb layer and the Ge substrate.

In Figure 5, we present the normalized PL signal from Ge-virgin and FLA-treated GePb samples under room temperature (a) and 77 K (b). For the measurements under room temperature (Figure 5a), the PL spectrum of the FLA-treated GePb sample exhibits a spectral broadening mainly toward the longer wavelength (red shift), indicating a bandgap engineering through Pb alloying, which can reduce the bandgap of Ge and shift the PL emission toward the longer wavelength [26]. However, the main PL emission peak is still near 1550 nm, indicating that the PL signal is still strongly influenced by the contributions of Ge substrate. This can be ascribed to the thickness of the Pb alloyed layer, which is less than 200 nm thick with much stronger defect-related nonradiative recombination than Ge substrate. The strong PL emission from the defect-free Ge substrate overlaps with the PL contribution from the GePb layer, making it challenging to distinguish but still shows a peak extension toward the longer wavelength. The signal decrease after 2060 nm is due to the responsivity cutoff of the InGaAs array detector used in the PL measurement system.

In Figure 5b, we compare the normalized PL spectra from the Ge-virgin sample and the FLA-treated GePb sample under 77 K. The direct-band transition near 1400 nm from both Ge and GePb layers is not dominant because carriers are frozen in the indirect-band L valley in the Ge conduction band. For the Ge-virgin sample at 77 K, the sharp PL peak at 1732 nm is attributed to the indirect-band transition from Ge, which shifts to 1700 nm in the GePb layer, possibly due to energy band modification from compressive strain conditions originating from the Ge/GePb interface where the Pb concentration is lower than the peak concentration. However, a new emission peak from 1800 to 2060 nm (detector cutoff) can be observed in our FLA-treated GePb sample, which is not observed in the pristine Ge sample. This provides evidence of Pb alloying into the Ge lattice and reducing the band gap of the L valley, making it a promising candidate for extended SWIR light source application.

The GePb-related PL emission cannot be resolved at room temperature, which can be attributed to the low PL intensity resulting from the limited thickness of the GePb layer and the strong overlap with the broad PL emission tail from the Ge substrate. This overlap leads to an extension of the overall PL spectrum toward longer wavelengths, but no distinct emission peak from GePb can be identified. As the temperature decreases, the PL contribution from the phonon sideband (PSB) of the Ge indirect bandgap transition is significantly reduced, and the zero-phonon line (ZPL) becomes narrower, in agreement with the Maxwell–Boltzmann probability distribution function [56]. As a result, the spectral overlap between the Ge and GePb emissions is minimized, allowing a distinct PL peak from GePb to be resolved.

**Figure 6:**
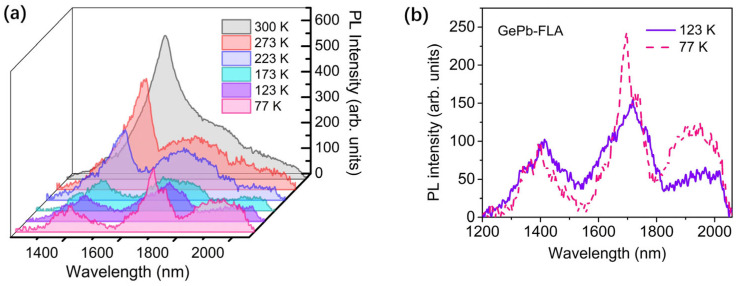
(**a**) Photoluminescence measurement of GePb-FLA sample at various temperatures. (**b**) The detailed PL comparison of the GePb sample at 123 K and 77 K.

Photoluminescence measurements of the GePb-FLA sample at various temperatures are shown in Figure 6a. As the measurement temperature decreases, the PL contribution from the direct bandgap transition of Ge substrate near 1550 nm shows both blue shift and significant intensity decreases due to carriers being frozen in the indirect band L valley of the Ge conduction band. The PL emission from the GePb layer becomes evident when the sample temperature drops below 123 K. This emission spectrum appears as a broad peak starting from 1800 nm and extending to the detector cutoff at 2060 nm, indicating bandgap modification via Pb alloying into the Ge lattice.

A detailed PL comparison of the GePb sample at 123 K and 77 K is shown in Figure 6b. With a temperature decrease from 123 K to 77 K, the PL emission peak between 1800 and 2060 nm shifts to shorter wavelengths, and the PL intensity increases as the temperature decreases, which is consistent with the behavior of the indirect-band transition in Ge [57], and the longer emission wavelength than virgin Ge also agrees well with the theorical predicted bandgap engineering of Pb alloyed in Ge [26], indicating that the PL emission peak between 1800 and 2060 nm might originate from the indirect-band transition of bandgap-engineered GePb material. The relatively larger full width at half maximum (FWHM) of the PL peak from the GePb layer can be attributed to inhomogeneous Pb concentration in the implanted layer, which typically follows a Gaussian distribution. The sharp decrease beyond 2060 nm is due to the cut-off of the PL measurement system.

We further discuss and aim to exclude the possibility that the PL signal originates from structures potentially present in our sample, such as Ge nanocrystals, polycrystalline Ge, and defects in Ge. This is achieved by comparing our PL results with previously reported PL characteristics from these structures:

1. Ge Nanocrystals: As reported in Refs. [58,59], Ge nanocrystals exhibit a higher bandgap than single-crystal Ge, which is particle-size-dependent. Their PL emission is strongly broadened, extending toward shorter wavelengths, and can cover the visible range up to the Ge bandgap at room temperature. In contrast, the photoluminescence observed in our work predominantly extends toward longer wavelengths, which is inconsistent with this origin.
2. Polycrystalline Ge: According to Ref. [56], the PL emission of polycrystalline Ge shifts slightly to shorter wavelengths compared to single-crystal Ge. While our GePb material contains polycrystalline Ge, no such blue-shifted PL spectra are observed in our results.
3. Defects in Ge: To the best of our knowledge, the only reported defect-related luminescence in Ge is threading dislocation luminescence, which produces a sharp PL peak at 2.4 μm and does not shift with temperature (Refs. [56,60,61]). Since this emission lies outside our measurement range, it cannot explain our PL results. Furthermore, most defect-related PL in semiconductors arises from fixed energy levels, producing narrow emission lines (Refs. [62,63]), which is not consistent with our observations. The new emission peak observed in our FLA-treated GePb sample is distributed around 1800–2000 nm at 77 K and shifts with temperature. This behavior cannot be attributed to defect-related luminescence.

## 4. Conclusions

In summary, we have shown the extension of the luminescence emission to longer wavelengths in Ge using a CMOS-compatible process involving Pb ion implantation and flash-lamp annealing. We fabricated GePb alloys integrated with the antireflective subwavelength nanostructure via ultra-fast solid-phase epitaxial regrowth. Photoluminescence spectroscopy at varying temperatures revealed bandgap modification and extended emission wavelengths in the GePb alloys. Notably, our ion-beam-implanted and flash-lamp-annealed GePb alloys exhibit two significant optical characteristics: reduced optical reflectance due to the subwavelength cavity structure and bandgap shrinkage toward longer wavelengths attributed to Pb alloying. These findings position flash-lamp-annealed GePb alloys as promising candidates for various optoelectronic applications.

## Data Availability

The original contributions presented in this study are included in the article. Further inquiries can be directed to the corresponding authors.

## Supplementary Materials

The following supporting information can be downloaded at: https://www.mdpi.com/article/10.3390/ma18102258/s1, Figure S1. Pb distribution profile simulated by the SRIM software. Figure S2. High-resolution TEM images of as-implanted GePb (a, b) and FLA-treated GePb (c, d). Panels (b) and (d) are enlarged views of the quadratic regions in panels (a) and (c), and indicate the amorphous structure of as-implanted GePb and the single-crystalline structure of FLA-treated GePb, respectively. The fast Fourier transforms of panels (b) and (d) are presented as the corresponding insets.
