# Supplementary material for: Optical Properties of GePb Alloy Realized by Ion Beam Technology
Source: Materials (Basel). 2025 May 13;18(10):2258. doi: 10.3390/ma18102258 (PMC12112875; doi:10.3390/ma18102258)
Supplement: Supplementary file 1 [file materials-18-02258-s001.zip › materials-3588213-supplementary.pdf]

Supplementary material

Optical properties of GePb alloy realized by ion beam tech-nology

Shuyu Wen [1,2,3], Yuan-Hao Zhu [1,3], Oliver Steuer [2,4], Mohd Saif Shaikh [2,5], Slawomir Prucnal [2], René Hübner [2], Andreas Worbs [2], Li He [1], Manfred Helm [2,6], Shengqiang Zhou [2], Jun-Wei Luo [1,3,*] and Yonder Berencén [2,*]

[1] State Key Laboratory of Semiconductor Physics and Chip Technologies, Institute of Semiconductors, Chinese Academy of Sciences, Beijing 100083, China; sywen@semi.ac.cn (S.W.); zhuyuanhao20@semi.ac.cn (Y.-H.Z.); heli2018@semi.ac.cn (L.H.)
[2] Helmholtz-Zentrum Dresden-Rossendorf, Institute of Ion Beam Physics and Materials Research, Bautzner Landstrasse 400, 01328 Dresden, Germany; o.steuer@hzdr.de (O.S.); m.shaikh@hzdr.de (M.S.S.); s.prucnal@hzdr.de (S.P.); r.huebner@hzdr.de (R.H.); a.worbs@hzdr.de (A.W.); m.helm@hzdr.de (M.H.); s.zhou@hzdr.de (S.Z.)
[3] Center of Materials Science and Optoelectronics Engineering, University of Chinese Academy of Sciences, Beijing 100049, China
[4] Institut für Werkstoffwissenschaft und Max-Bergmann-Zentrum für Biomaterialien, TUD Technische Universität Dresden, 01062 Dresden, Germany
[5] Faculty of Electrical and Computer Engineering, Technische Universität Dresden, Helmholtzstraße 18, 01069 Dresden, Germany
[6] Institute of Applied Physics, Technische Universität Dresden, 01062 Dresden, Germany
* Correspondence: jwluo@semi.ac.cn (J.-W.L.); y.berencen@hzdr.de (Y.B.)

SRIM simulation and Pb concentration estimation: In this study, we employed Pb implantation with a fluence of 2×10^{15} cm^{-2} and an energy of 200 keV. The theoretical Pb distribution profile, simulated using SRIM software[34], is shown in Fig. S1. According to the simulation, ideal Pb implantation yields a 60 nm-thick GePb layer with a maximum Pb concentration of approximately 2%. However, due to the formation of a cavity structure during the Pb implantation, Pb atoms are distributed across both the GePb cavities and a homogeneous GePb layer located much deeper than predicted by SRIM. This divergence complicates the accurate estimation of the Pb concentration.

In this case, we estimated a filling factor of 50% within the 130 nm-thick cavity structures based on SEM and TEM images. This corresponds to the same volume of Ge lattice as a 65 nm-thick GePb layer without cavities. Pb atoms were also distributed in the 60 nm-thick homogeneous GePb layer beneath the cavity structures, as shown in the TEM images. Therefore, the total Pb atoms are effectively distributed within an equivalent 125 nm-thick homogeneous Ge layer -- nearly double the thickness predicted by the SRIM simulation -- reducing the peak Pb concentration by half, to approximately 1%, in the as-implanted sample. Furthermore, by comparing the RBS signals of Pb in the as-implanted sample and FLA-treated samples, we observed a Pb loss of approximately 30% after FLA, likely due to Pb out-diffusion and removal during HF

etching. Consequently, we estimate a peak Pb concentration of 0.7% following FLA treatment.

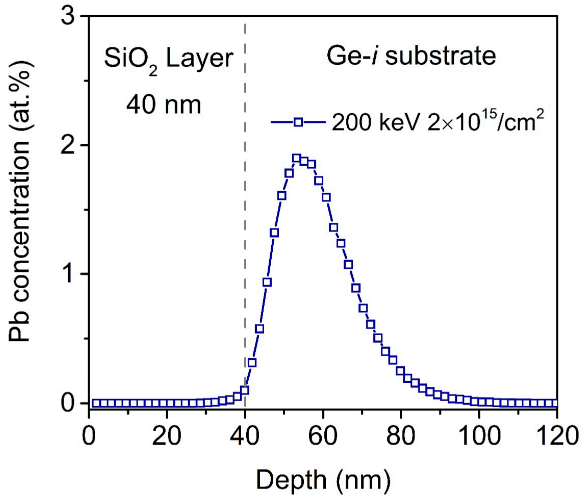

Fig.S1 Pb distribution profile simulated by the SRIM software

TEM: High-resolution TEM images of the as-implanted and FLA-treated GePb samples are shown in Fig. S2 (a) and (c), respectively. Enlarged views of the highlighted regions are presented in panels (b) and (d) with insets displaying the corresponding fast Fourier transforms. Pb ion implantation results in complete amorphization of the near-surface region, as seen in Fig. S2(b). In contrast, the FLA-treated sample exhibits clear signs of epitaxial regrowth, as shown in Fig. S2(d).

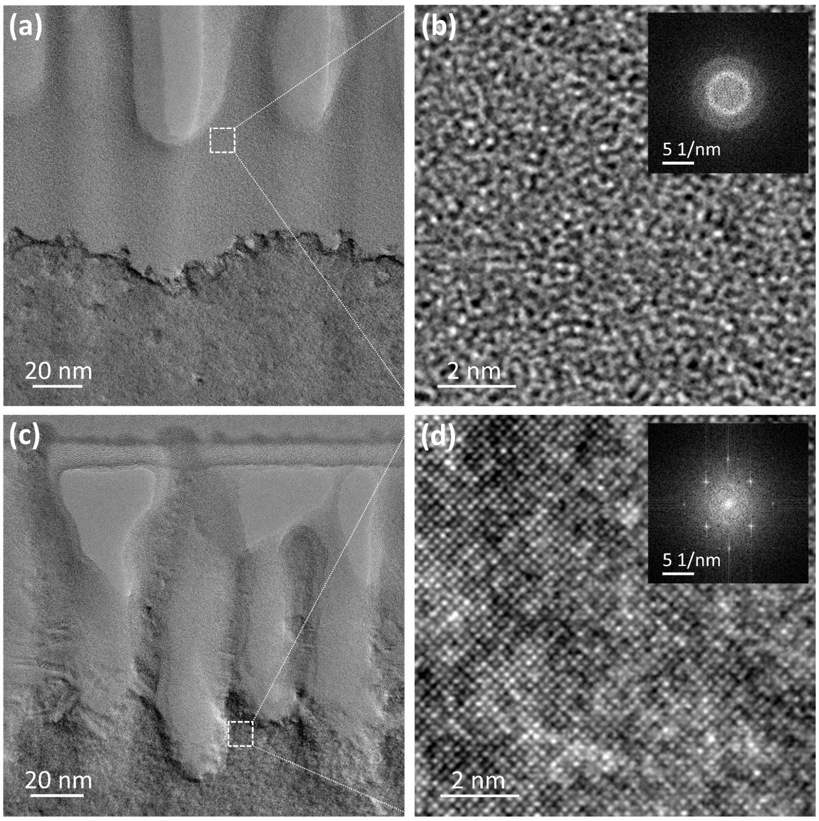

Figure S2. High-resolution TEM images of as-implanted GePb (a, b) and FLA-treated GePb (c, d). Panels (b) and (d) are enlarged views of the quadratic regions in panels (a) and (c), and indicate the amorphous structure of as-implanted GePb and the single-crystalline structure of FLA-treated GePb, respectively. The fast Fourier transforms of panels (b) and (d) are presented as the corresponding insets.

Reference

[1] Ziegler, James F., Matthias D. Ziegler, and Jochen P. Biersack. "SRIM–The stopping and range of ions in matter (2010)." *Nuclear Instruments and Methods in Physics Research Section B: Beam Interactions with Materials and Atoms* **2010**, 268.11-12,1818-1823, doi.org/10.1016/j.nimb.2010.02.091
